# Multi-omics profiling reveal responses of three major *Dendrobium* species from different growth years to medicinal components

**DOI:** 10.3389/fpls.2024.1333989

**Published:** 2024-02-23

**Authors:** Yingdan Yuan, Jiajia Zuo, Xin Wan, Runyang Zhou, Wei Xing, Sian Liu

**Affiliations:** ^1^ College of Horticulture and Landscape Architecture, Yangzhou University, Yangzhou, China; ^2^ Jiangsu Academy of Forestry, Nanjing, China; ^3^ Jiangsu Yangzhou Urban Forest Ecosystem National Observation and Research Station, Yangzhou, China

**Keywords:** *Dendrobium*, transcriptome, metabolome, microbiome, flavonoid

## Abstract

*Dendrobium* is a perennial herb found in Asia that is known for its medicinal and ornamental properties. Studies have shown that the stem is the primary medicinal component of *Dendrobium* spp. To investigate the effect of the species and age of *Dendrobium* (in years) on the content of its medicinal components, we collected the stems of 1-to-4-year-old *D. officinale*, *D. moniliforme*, and *D. huoshanense*, sequenced the transcriptome, metabolome, and microbiome, and analyzed the data in a comprehensive multi-omics study. We identified 10,426 differentially expressed genes (DEGs) with 644 differentially accumulated metabolites (DAMs) from 12 comparative groups and mapped the flavonoid pathway based on DEGs and DAMs. Transcriptomic and metabolomic data indicated a general trend of the accumulation of flavonoids exhibiting pharmacological effects in the three *Dendrobium* species. In addition, joint metabolome and microbiome analyses showed that actinobacteria was closely associated with flavonoid synthesis with increasing age. Our findings provide novel insights into the interactions of flavonoids of *Dendrobium* with the transcriptome and microbiome.

## 1 Introduction

*Dendrobium* is a perennial herb valued as an herbal medicine. It was first identified by Olaf Swartz in 1799 (Puchooa, 2004). It belongs to the second-largest genus in the orchid family. There are several species of *Dendrobium*, and the *Dendrobium* genera discovered thus far include approximately 1100 species, among which 74 species are found in subtropical China, including *D. huoshanense*, *D. nobile*, *D. fimhriatum*, and *D. officinale* (Pharmacopoeia, 2010; Deng et al., 2018). In China, *Dendrobium* has a long history of medicinal use and has proven hypoglycemic, hypolipidemic, antitumor, pain-relieving, and antipyretic effects (Teoh, 2016). The chemical components of *Dendrobium* spp. are diverse, some of which include polysaccharides, alkaloids, bibenzoids, phenanthrenes, sesquiterpenes, and inulinones (Chen, 2013). The key medicinal components are polysaccharides, alkaloids, and flavonoids, which impart the unique pharmacological properties of *Dendrobium* (Wang et al., 2021).

With the development of high-throughput sequencing technologies, multi-omics studies have deepened our understanding of molecular mechanisms underlying the synthesis of the major components of medicinal plants. At present, multi-omics studies of *Dendrobium* primarily focus on the combined transcriptome and metabolome analysis. Combined transcriptomics and metabolomics analysis is an important tool for identifying variations in metabolites and gene regulation mechanisms in different tissues of different plants. The method is suitable for establishing synergistic networks for active ingredient biosynthesis and exploring key enzyme-synthesizing genes involved in metabolite synthesis (Trikka et al., 2015). Zhan et al. found that flavanone 3-hydroxylase (*F3H*) and leucoanthocyanidin dioxygenase (*LDOX*) were the key genes responsible for color changes in purple *D. officinale* (Zhan et al., 2020). Zhang et al. used multi-omics methods to determine the adaptation of *D. officinale* leaf tissue to salt stress response through the enhanced synthesis of secondary metabolites (Zhang et al., 2021b). Wu et al. used transcriptomic and metabolomic techniques based on physiological characterization to determine the response to low temperature domestication during the growth of *D. officinale* (Wu et al., 2016). Zhang et al. showed that purine and phenylpropanoid biosynthetic pathway may play an important role in the response to drought stress in *D. sinense* (Zhang et al., 2021a). Overall, multi-omics studies on *Dendrobium* spp. primarily focus on stress responses, growth and development, and secondary metabolism, with *D. officinale* being the most studied species.

Several factors, such as rainfall, temperature, light, humidity, and other abiotic factors, and biotic factors such as insects, herbivores, and microorganisms, affect the synthesis of plant secondary metabolites (Borges et al., 2017). Researchers have identified an inextricable link between the plant microbiome and secondary metabolism, especially the endophytic bacteria in plants (Etalo et al., 2018). In brief, plant metabolites can shape the microbiome of plants, which can influence the synthesis of related metabolites in plants or even directly synthesize metabolites in plants (Jacoby et al., 2021; Koprivova and Kopriva, 2022). For example, the endophytic fungus *Taxomycesan dreanae* in *Taxus brevifolia* can synthesize paclitaxel, a key anticancer substance in *Taxus* plants (Stierle et al., 1993). Many bacteria can act as inducers to enhance the biosynthesis of secondary metabolites, such as alkaloids, flavonoids, and terpenoids, in medicinal plants (Chamkhi et al., 2021). At present, studies on the Dendrobium microbiome primarily focus on the composition and diversity of microbial communities. Few studies have analyzed the metabolome and microbiome collectively (Chen et al., 2020; Zhao et al., 2023). Here, we aimed to use an integrated transcriptome-metabolome-microbiome analysis method to further elucidate the reciprocal regulatory relationship among host gene expression, microbial composition, and metabolites.

In this study, three annual, biennial, triennial, and quadrennial plants each from the species *D. huoshanense*, *D. officinale*, and *D. moniliforme* were collected. The metabolites of *Dendrobium* stems were identified by widely targeted metabolomics techniques. Transcripts from the stem samples were sequenced by RNA sequencing, and the endophytess of the stems were sequenced by amplicon 16S and internal transcribed spacer (ITS) rRNA. We established the metabolic profiles, transcripts, and microbial communities of the stems of the different *Dendrobium* species with different growth years, described the synthetic pathways of important secondary metabolites of *Dendrobium*, screened key genes in the synthetic pathways of important secondary metabolites of *Dendrobium*, and explored the microbial composition among endophytic bacteria in *Dendrobium* stems. This study focused on the following key factors: (1) differences in the composition of major secondary metabolites in different species of *Dendrobium*, and whether the accumulation patterns of these secondary metabolites are related to the expression of corresponding key genes; (2) the effects of age and species on the accumulation of major medicinal components of *Dendrobium*; (3) and the exploration of potential microbiota affecting the synthesis of major medicinal components of *Dendrobium*. Our findings are relevant for cultivation and research on *Dendrobium*.

## 2 Materials and methods

### 2.1 Plant material and sample collection

**Figure 1:**
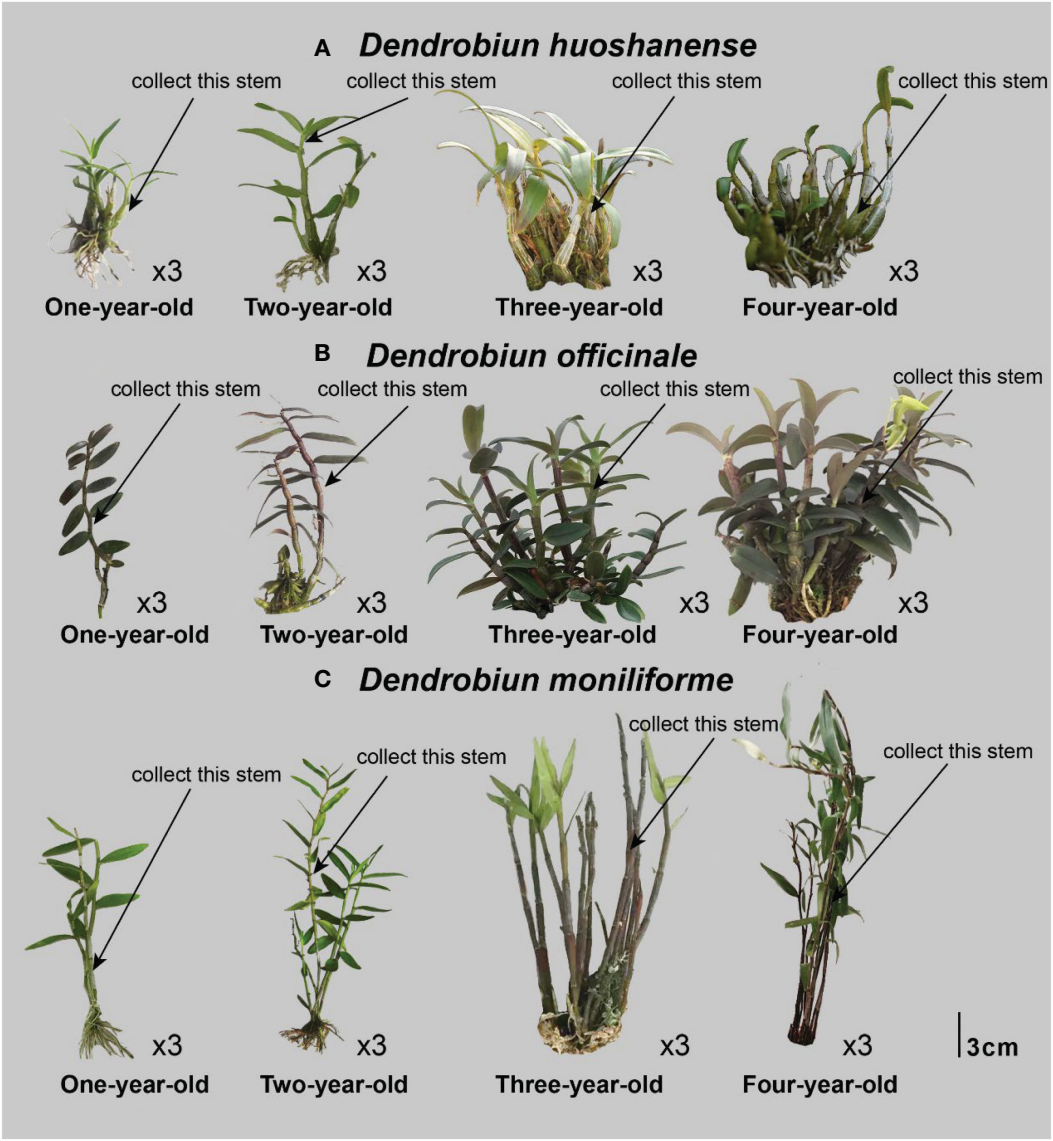
Stem samples collected from *Dendrobium* of different years. **(A)** Dendrobiun huoshanense; **(B)** Dendrobiun officinale; **(C)** Dendrobiun moniliforme.

Three different *Dendrobium* species (*D. huoshanense*, *D. officinale*, and *D. moniliforme*) were cultivated artificially and housed in a greenhouse at Anhui Tongjisheng Biotechnology Co., Ltd. The cultivation conditions for the samples were similar to those used in our previous study (Yuan et al., 2019). Plant stems from one-year *D. huoshanense* (1Dh_1, 1Dh_2, 1Dh_3), *D. officinale* (1Do_1, 1Do_2, 1Do_3), and *D. moniliforme* (1Dm_1, 1Dm_2, 1Dm_3); two-year *D. huoshanense* (2Dh_1, 2Dh_2, 2Dh_3), *D. officinale* (2Do_1, 2Do_2, 2Do_3), and *D. moniliforme* (2Dm_1, 2Dm_2, 2Dm_3); three-year *D. huoshanense* (3Dh_1, 3Dh_2, 3Dh_3), *D. officinale* (3Do_1, 3Do_2, 3Do_3), and *D. moniliforme* (3Dm_1, 3Dm_2, 3Dm_3); and four-year *D. huoshanense* (4Dh_1, 4Dh_2, 4Dh_3), *D. officinale* (4Do_1, 4Do_2, 4Do_3), and *D. moniliforme* (4Dm_1, 4Dm_2, 4Dm_3) were used for the study. We selected the first and second sections of the uppermost main stems of *Dendrobium* at each instance (**Figure 1**), for specific picking locations. To extract RNA, DNA, and metabolites, a part of the material was frozen in liquid nitrogen at -80°C. Before the material was used for evaluating total alkaloids and flavonoids, it was washed and dried. In the current study, we included three biological replicates for all tests.

### 2.2 Widely-targeted metabolomic profiling

To investigate metabolite variations in the different stem samples, we conducted widely targeted metabolomics experiments on samples with three biological duplicates for each growth year and species. Metware Biotechnology Ltd. conducted metabolite analysis of the stem samples (Wuhan, China). The Analyst 1.6.3 program was used to analyze metabolic data. The differences between the metabolites of the two samples were maximized using OPLS-DA to identify differential metabolites (orthogonal projections to latent structures-discriminant analysis). Based on the OPLS-DA results, the derived Variable Importance in Projection (VIP) of the OPLS-DA model for multivariate analysis was used to conduct a preliminary screening of differential metabolites. Foldchange ≥ 2, foldchange ≤ 0.5, and VIP ≥ 1 were the differential metabolites in our inquiry for the next step of the analysis. Using R and principal component analysis (PCA), we examined the accumulation of *Dendrobium* metabolites in different growth years. All samples were evaluated using a cluster heatmap developed once the data were standardized.

### 2.3 Total RNA extraction, transcriptome sequencing, and differential expression analysis

Total RNA was extracted from the stem tissue samples. The complete frozen stems were crushed in liquid nitrogen, and RNA was extracted using an Omni Plant RNA kit (CWBIO, China) and checked for purity. A TruSeq™ RNA sample preparation kit (Illumina, USA) was used to prepare the cDNA library for each sample. The cDNA libraries were sequenced at high throughput using the Illumina HiSeq 2500 platform. The raw data of all transcriptomes were stored in the NCBI database under the codes PRJCA007253, PRJNA776680, and PRJNA776418.

Cutadapt was used to filter the data and obtain clean reads (Martin, 2011). The filtered clean reads were mapped to the reference genome using HISAT2 (http://dx.doi.org/10.1038/srep19029) (Sirén et al., 2014; Zhang et al., 2016a). Gene expression levels were assessed using Fragments Per Kilobase Million (FPKM) values. Genes showing an absolute log2 fold change (FC) > 1 with a P-value < 0.05 were considered to be differentially expressed in *D. huoshanense*, *D. officinale*, and *D. moniliforme*. TopGo and clusterProfiler packages were used for Gene Ontology (GO) and Kyoto Encyclopedia of Genes and Genomes (KEGG) enrichment analysis of the annotated differentially expressed genes (DEGs), respectively.

### 2.4 DNA extraction, high-throughput sequencing, and microbial data analysis

Microbial DNA from 72 samples (three species of 1-to-4-year-old *Dendrobium*, with triplicates of each species of *Dendrobium* with different growth years) was extracted using the CTAB/SDS method (Niemi et al., 2001). The DNA concentration and purity were tested on a 1% agarose gel. Depending on the concentration, DNA was diluted to 1 ng/μL with sterile water. Primer sets 515F (5′-GTGCCAGCMGCCGCGG-3′) and 806R (5′-GGACTACHVGGGTWTCTAAT-3′) were used to generate bacterial libraries with a unique 6-nt barcode at 5’ of the forward primer to amplify the V4 region of the 16S rRNA gene for each sample. Similar to the process used for constructing the bacterial library, the ITS1 region of the fungus was amplified using ITS1-1F-F (CTTGGTCATTTAGGAAGTAA) and ITS1-1F-R (GCTGCGTTCTTCATCGATGC) (Caporaso et al., 2012). Phusion® High-Fidelity PCR Master Mix (New England Biolabs) was used for PCR. Next, the PCR products were mixed with an equal volume of 1× loading buffer (containing SYB green), and their purity was tested using 2% agarose gel electrophoresis. PCR products were mixed at equal density ratios. The mixed PCR products were purified using the GeneJETTM Gel Extraction Kit (Thermo Scientific). Sequencing libraries were generated and index codes were added using the TruSeq® DNA PCR-Free Sample Preparation Kit (Illumina) according to the manufacturer’s recommendations. The library quality was evaluated on a Qubit^@^ 2.0 fluorometer (Thermo Scientific) and an Agilent Bioanalyzer 2100 system. After purification and quantification, the libraries were sequenced on an Illumina NovaSeq 6000 platform according to standard protocols.

Quality control processing of raw data from the 16S V4 bacterial and fungal ITS1 regions was performed using QIIME (V1.9.1, http://qiime.org/scripts/split libraries fastq.html) (Caporaso et al., 2010). Paired reads were processed using FLASH (V1.2.7, http://ccb.jhu.edu/software/FLASH/) (Magoč and Salzberg, 2011). After quality control, bacteria and fungi were annotated by matching the Silva sequences with the UCHIME algorithm and the Unite database (ITS: http://unite.ut.ee/) (UCHIME, http://www.drive5.com/usearch/manual/uchime algo.html) (Quast et al., 2012; Kõljalg et al., 2013). The calculation was performed using Usearch and Vsearch to assign sequences to the same amplicon sequence variants (ASVs) (Edgar, 2010; Rognes et al., 2016).

### 2.5 Statistical analysis

Quantitative data were statistically analyzed using the SPSS software (version 25.0; SPSS Inc., Chicago. IL, USA) using one-way ANOVA to determine significant differences at a p-value of > 0.05. If significant differences were observed, Duncan’s *post-hoc* test was performed to determine the values that differed from all other values. An infrared spectrogram was drawn using Origin 2019b (OriginLab, Northampton. MA, USA). Other analyses were conducted and figures were constructed using R (version 3.6.1; https://www.r-project.org/). The “dplyr” software was used for data cleaning. “vegan,” “phyloseq,” and “ggplot2” packages were used to calculate alpha diversity indexes such as Shannon, Pielou, Chao1, and ACE (McMurdie and Holmes, 2013; Oksanen, 2013; Wickham, 2011). Beta diversity was calculated using the “phyloseq” 154 and visualized using “ggplot2.” Histograms of the top 10 relative abundances at the phylum and genus levels were generated using “ggplot2.” The “ggClusterNet” package was used to create correlation network diagrams of bacterial communities (https://github.com/taowenmicro/ggClusterNet).

## 3 Results

### 3.1 Comparative transcriptomics of three *Dendrobium* species with different growth years

We used transcriptome sequencing to analyze the differential expression of genes in the stems of three 1-to-4-year-old *Dendrobium* cultivars. The screening criteria for DEGs were crucial. We identified DEGs based on of |log2 (fold change)| > 1 and P value < 0.05. Owing to the large number of comparison groups among the 36 samples, we only focus on the DEGs of the stems with the same growth age from the three cultivars of *Dendrobium*. Twelve comparison groups were constructed in this study as follows: 1Dh vs. 1Dm, 1Dh vs. 1Do, 1Do vs. 1Dm, 2Dh vs. 2Dm, 2Dh vs. 2Do, 2Do vs. 2Dm, 3Dh vs. 3Dm, 3Dh vs. 3Do, 3Do vs. 3Dm, 4Dh vs. 4Dm, 4Dh vs. 4Do, and 4Do vs. 4Dm. A total of 10,426 DEGs were identified in 12 comparison groups. The 2Do vs. 2Dm comparison group had the highest number of DEGs, with 1665 upregulated and 2900 downregulated DEGs. In contrast, the 2Dh vs. 2Dm comparison group had the fewest DEGs, with 264 up-regulated and 345 down-regulated DEGs (**Supplementary File Figure S1**).

**Figure 2:**
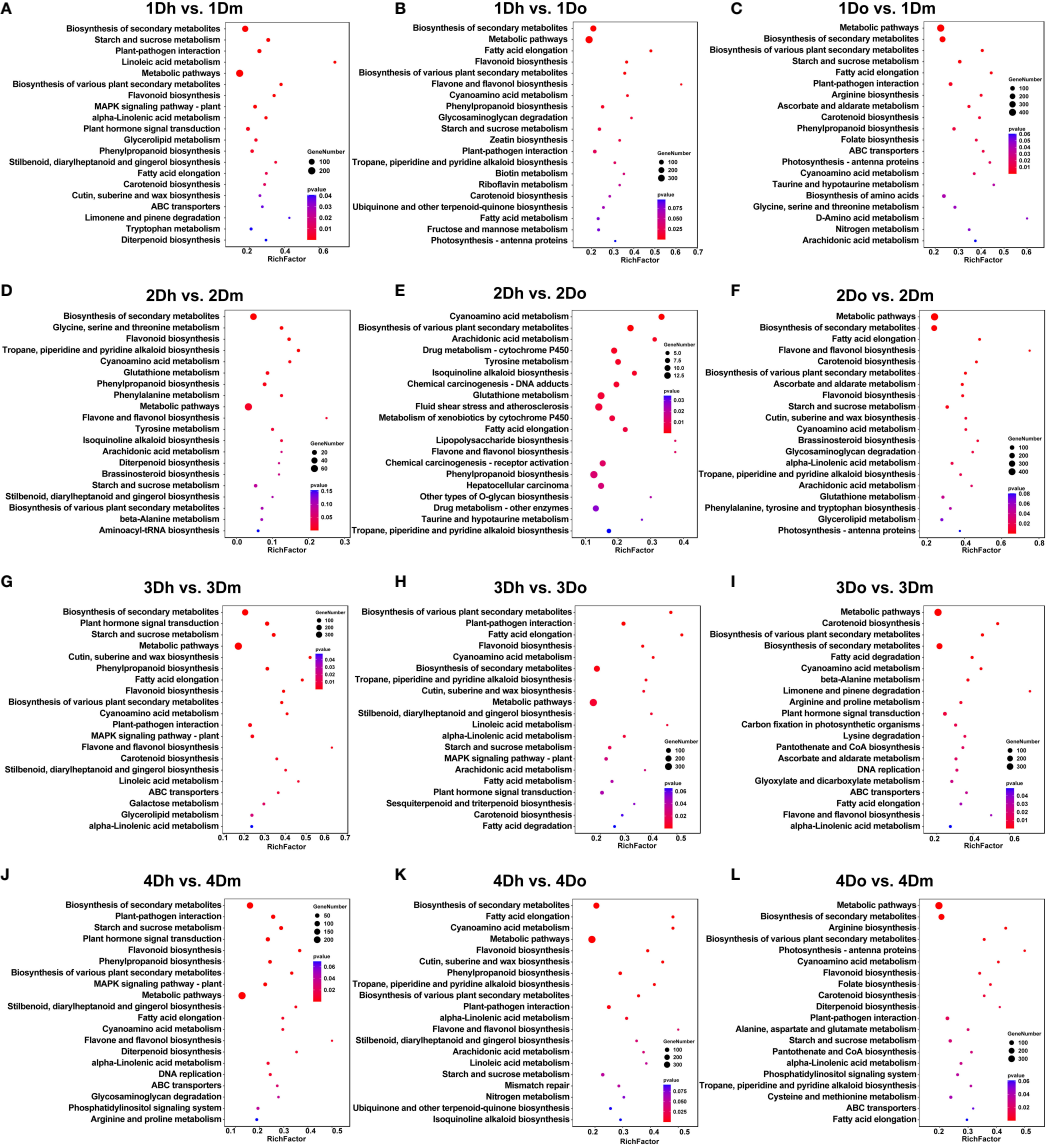
The KEGG pathway analysis of DEGs. **(A)** 1Dh vs 1Dm; **(B)** 1Dh vs 1Do; **(C)** 1Do vs 1Dm; **(D)** 2Dh vs 2Dm; **(E)** 2Dh vs 2Do; **(F)** 2Do vs 2Dm; **(G)** 3Dh vs 3Dm; **(H)** 3Dh vs 3Do; **(I)** 3Do vs 3Dm; **(J)** 4Dh vs 4Dm; **(K)** 4Dh vs 4Do; **(L)** 4Do vs 4Dm.

To further analyze the biological function of DEGs, we performed KEGG enrichment analysis. The biosynthesis of secondary metabolites is significantly enriched in all comparison groups, indicating that genes related to secondary metabolites play a key regulatory role in the stems of the three *Dendrobium* species (**Figure 2**). Since secondary metabolites were the active components of many medicinal plants, we would next focus on the synthetic pathways associated with secondary metabolites. Phenylpropanoid biosynthesis was significantly enriched in several comparison groups except for the four comparison groups: 2Do vs. 2Dm, 3Dh vs. 3Do, 3Do vs. 3Dm, 4Do vs. 4Dm (**Figure 2**). As one of the important secondary metabolic pathways of plants, phenylpropane metabolism can produce more than 8,000 metabolites such as lignin, flavonoids and phenolic acids. In addition, we found that flavonoid biosynthesis downstream of the phenylpropyl pathway was significantly enriched in the comparison groups of 1Dh vs. 1Dm, 1Dh vs. 1Do, 2Dh vs. 2Dm, 2Do vs. 3Dm, 3Dh vs. 3Dm, 3Dh vs. 3Do, 4Dh vs. 4Dm, 4Dh vs. 4Do and 4Do vs. 4Dm. Flavone and flavonol biosynthesis was significantly enriched in the comparison group of 1Dh vs. 1Do, 2Dh vs. 2Dm, 2Dh vs. 2Do, 3Dh vs. 3Dm, 3Do vs. 3Dm, 4Dh vs. 4Dm, 4Dh vs. 4Do. Meanwhile, tropane, piperidine and pyridine alkaloid biosynthesis in the comparison group 1Dh vs. 1Do, 2Dh vs. 2Dm, 2Dh vs. 2Do, 2Do vs. 2Dm, 3Dh vs. 3Do, 4Dh vs. 4Do, 4Do vs. 4Dm were significantly enriched. These results indicated that genes related to the synthesis of flavonoids and alkaloids played key regulatory roles in the stems of three *Dendrobium* species. Flavonoids and alkaloids are one of the main medicinal components of *Dendrobium*, therefore, we focus on these two metabolites in the metabolome analysis below.

### 3.2 Comparative metabolomics of three *Dendrobium* species in different years

Based on Fold change ≥ 2, fold change ≤ 0.5, and VIP ≥ 1, we identified 644 DAMs in 12 comparison groups (**Supplementary File Figure S2**). The number of DAMs in the 2Do vs. 2Dm comparison group was the highest (310), whereas the number of DAMs in the 4Dh vs. 4Dm comparison group was the lowest (199). To further explore the function of these DAMs, KEGG enrichment analysis was conducted (**Supplementary File Figure S3**). Flavone and flavonol biosynthesis were significantly enriched in all comparison groups. Flavonoid biosynthesis was enriched in the comparison group of 1Dh vs. 1Dm, 3Dh vs. 3Do, 3Do vs. 3Dm, 4Dh vs. 4Dm. It indicated that the content of flavonoids especially flavones and flavonols in the three kinds of stems of Dendrobium may vary widely. Tropane, piperidine, and pyridine alkaloid biosynthesis were significantly enriched only in two comparison groups: 2Dh vs. 2Do and 2Do vs. 2Dm. Therefore, we focused on flavonoid biosynthesis in our subsequent combined transcriptome-metabolome analysis.

**Figure 3:**
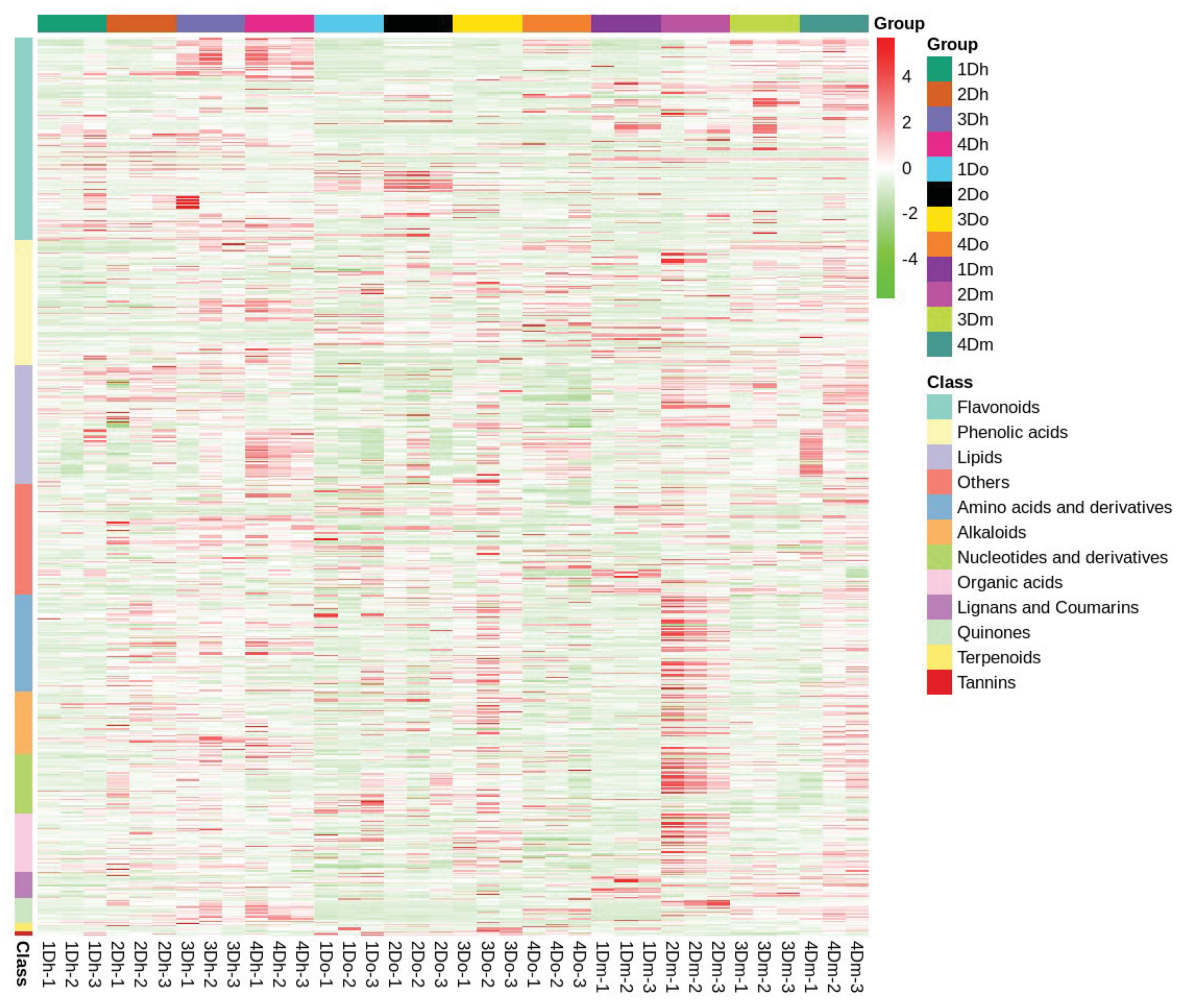
Cluster heatmap of metabolite abundance based on hierarchical cluster analysis.

Using LC-MS analysis, we identified 767 compounds, including 173 flavonoids (65 flavonoids, 42 flavonols, 3 chalcones, 12 dihydroflavonoids, 7 dihydroflavonols, 4 isoflavones, 4 flavanols, and 36 flavonoid carboglycosides), systematically and comprehensively identified the composition of flavonoids in the stems of the three *Dendrobium* species, and confirmed that the stems of *D. huoshanense*, *D. officinale*, and *D. moniliforme* were rich in flavonoids. Further, based on hierarchical cluster analysis, a heatmap of metabolite abundance clustering was drawn. The heatmap revealed an upward trend in the relative expression of most flavonoids in *D. huoshanense* and *D. moniliforme* as the years progressed (**Figure 3**). In *D. officinale*, the relative expression of most flavonoids reached the highest in the second year. In *D. huoshanense*, the relative expressions of phenolic acids, lignans and coumarins and quinones increased with age. In *D. officinale*, except flavonoids and quinones, the relative expression of other metabolites reached the highest in the third year. In *D. moniliforme*, the relative expression levels of most metabolites, such as lipids, organic acids, quinones and terpenoids, reached their maximum in the second year and then gradually decreased. The findings suggest that the accumulation pattern of metabolites varies among different varieties of *Dendrobium*, potentially leading to a decline in the content of certain medicinal ingredients as the plant ages.

### 3.3 Changes in metabolites and genes related to flavonoid biosynthesis

**Figure 4:**
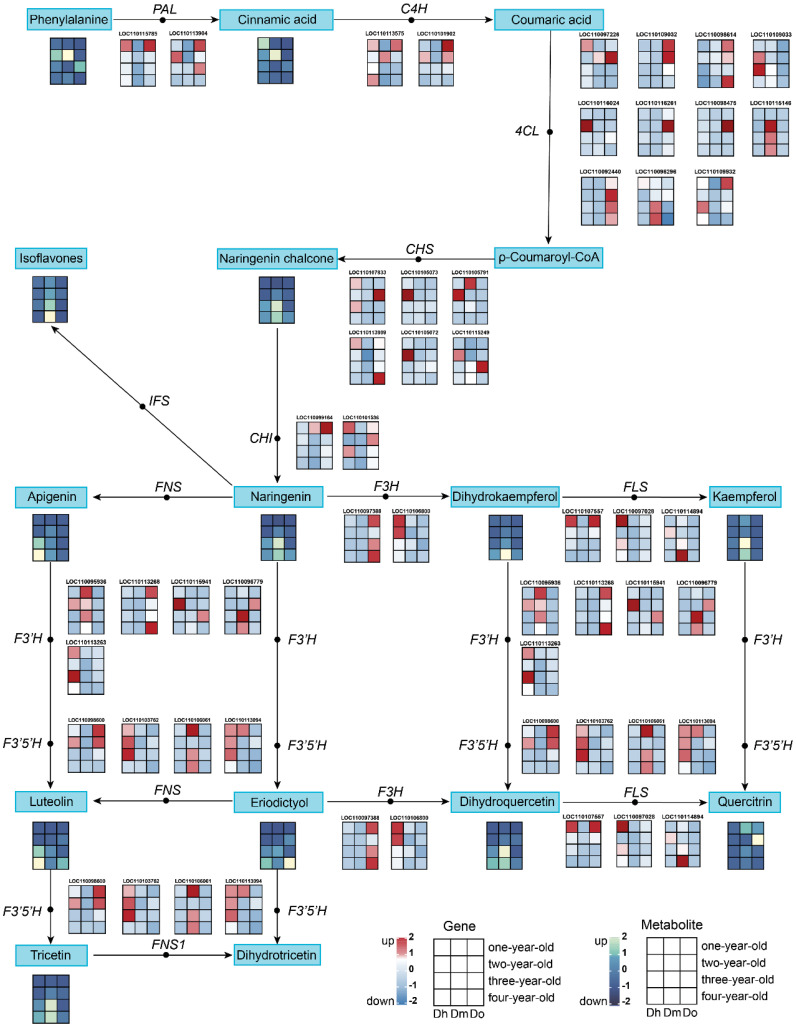
The pathway associated with flavonoid biosynthesis. The expression heatmap of the key metabolites and transcripts for flavonoid synthesis between four years of three *Dendrobium* species was shown by the colored cell on the bottom. Key enzyme gene abbreviation: *PAL*, phenylalanine ammonia lyase; *4CL*, 4-coumarate: CoA ligase; *C4H*, cinnamate 4-hydroxylase; *CHS*, chalcone synthase; *CHI*, chalcone isomerase; *IFS*, isoflavone synthase; *F3H*, flavonoid 3-hydroxylase; *F3’H*, flavonoid 3’-hydroxylase; *F3’5’H*, flavonoid 3’,5’-hydroxylase; *FNS*, flavone synthase; *FNS1*, flavone synthase I; *FLS*, flavonol synthase.

KEGG enrichment analysis of the transcriptome and metabolome showed that the DEGs and DAMs of the three *Dendrobium* species with different growth years were associated with flavonoid biosynthesis. We further analyzed the expression of related metabolites and genes involved in flavonoid biosynthesis in the selected *Dendrobium* species. We identified 37 DEGs associated with flavonoid biosynthesis in the transcriptome data (**Figure 4**). In all, we identified phenylalanine, cinnamic acid, naringenin chalcone, naringenin, isoflavones, apigenin, dihydrokaempferol, kaempferol, dihydroquercetin, quercitrin, eriodictyol, luteolin, and tricetin as 13 DAMs related to the flavonoid biosynthetic pathway in the metabolomic data from the samples.

The results showed that the relative expression levels of most flavonoids, such as naringenin, isoflavones, apigenin, luteolin, and tricetin, in *D. huoshanense* and *D. moniliforme* were higher in the third and fourth years. This is consistent with the trend observed in the relative expression of structural genes downstream of the flavonoid synthesis pathway. In *D. officinale*, the relative expression levels of many flavonoids (such as isoflavones, tricetin, quercitrin, and dihydroquercetin) peaked in the second year. This was consistent with the expression trend of structural genes upstream of the flavonoid biosynthesis pathway in *D. officinale*. The expression of *PAL*, *C4H*, *4CL*, *CHI*, and other genes was higher in the first- and second-year stems. When comparing the relative expression of flavonoids in the stems of different kinds of *Dendrobium* of the same growing age, we found that compared with the other two kinds of *Dendrobium*, the relative contents of isoflavones, naringenin chalcone, naringenin, kaempferol, tricetin were the highest in *D. moniliforme*. This shows the potential of *D. moniliforme* as a medicinal plant in addition to an ornamental plant.

### 3.4 Taxonomic characteristics of endophytic microbes of the three *Dendrobium* species

**Figure 5:**
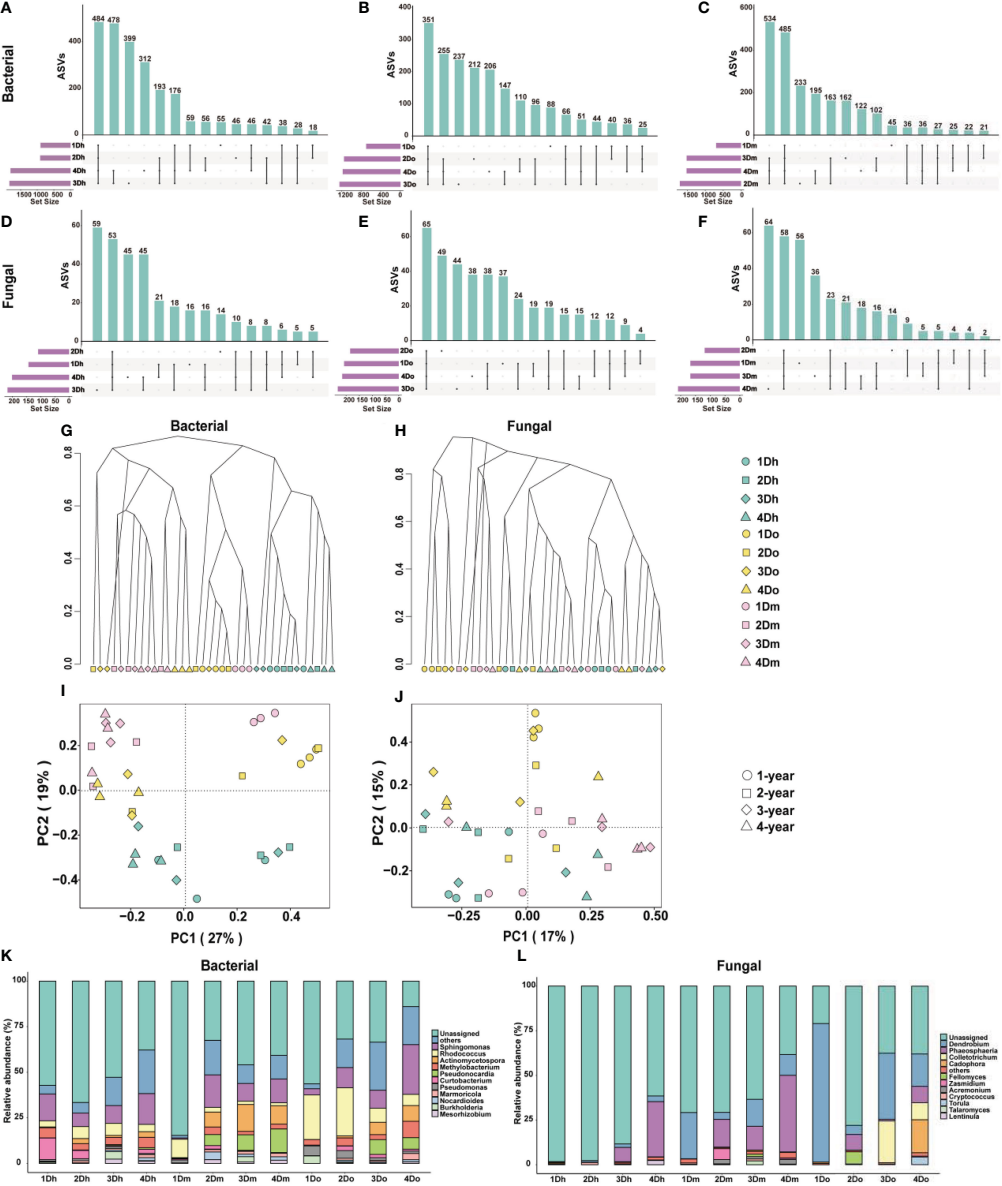
Microbial community differences in three kinds of *Dendrobium* of 1–4-year-old. **(A–F)** Upset diagram representing bacterial **(A–C)** and fungal **(D–F)** amplicon sequence variants (ASVs) associated with the endophytes from the stems of three kinds of *Dendrobium* of 1-4-year-old. **(G, H)** Hierarchical clustering of bacterial **(G)** and fungal **(H)** communities showing the Bray-Curtis dissimilarity of the whole ASV count data. **(I, J)** Principal component analysis (PCA) of bacterial **(I)** and fungal **(J)** communities. **(K, L)** Top 10 relative abundances of bacterial **(K)** and fungal **(L)** communities classified at genus.

Many studies have shown that the accumulation of secondary metabolites in medicinal plants is closely related to the metabolism of endophytes. To investigate the community composition of endophytes in the stems of three *Dendrobium* species aged 1-4 year, 16S rRNA and ITS sequencing analyses were used to analyze the stem samples. We used an Upset diagram to show the number of ASVs shared by endophytic bacteria in the stems of *Dendrobium* of the same variety and different years, where **Figures 5A–C** is bacteria and **Figures 5D–F** is fungi. The results showed that each *Dendrobium* had a large number of unique ASVs, indicating that the endophytes in the stems of the three *Dendrobium* species were very different.

To compare the diversity of the microbial communities of the endophytes of the stems, we performed the α-diversity analysis, and the results are shown in **Supplementary File Tables S1**, **S2**. The results showed that the differences in the diversity of the fungal communities in the samples were not significant, whereas the bacterial communities were more diverse. Shannon, Chao1, and ACE indices indicated that the alpha diversity of two-year-old *D. moniliforme* was significantly higher than that of one-year-old *D. moniliforme* and *D. officinale*, indicating that the diversity of the bacterial community in two-year-old *D. moniliforme* was significantly higher than that of one-year-old *D. moniliforme* and *D. officinale*. In addition, the alpha diversity of two-year-old *D. moniliforme* was the highest among all samples, indicating that two-year-old *D. moniliforme* had the highest bacterial community diversity. To compare bacterial and fungal community compositions between samples, we calculated the Bray-Curtis metric, which is related to beta diversity. Hierarchical clusters of bacteria showed that samples of the same species were mostly clustered together, whereas samples of different species and the same age were in close proximity (**Figure 5G**). Hierarchical clusters of the fungus showed the evolutionary distance of the phylogeny between one-year-old *D. officinale* and other samples (**Figure 5H**). In the PCoA figure for bacteria, PCo1 and PCo2 accounted for 27% and 19% of the total variance, respectively (**Figure 5I**). Meanwhile, one-year-old *D. moniliforme* and *D. officinale* were positioned farther away from the other samples, indicating that the endophytic communities in their stems were more different from each other. In the PCoA of fungi (**Figure 5J**), PCo1 and PCo2 accounted for 17% and 15% of the total variance, respectively. The differences between the endophytic bacterial communities in the stems of different *Dendrobium* species (aged from 1 to 4 years) were greater than the differences between the endophytic fungal communities.

Taxonomy at the genus level showed different patterns between bacterial and fungal communities in different samples, with different compositions of endophytic communities in stems of the same species but different growth years. As shown in **Figure 5K**, in terms of bacterial community composition, *actinomycetospora* accounted for a higher proportion of endophytic bacterial community in *D. moniliforme* compared to the other two *Dendrobium* species. The proportion of *pseudonocardia* in the endophytic bacterial community of *D. moniliforme* gradually increased with age. Among all the samples, *curtobacterium* occupies the largest proportion in 1Dh. The proportion of *rhodococcus* in the three species of *Dendrobium* showed a decreasing trend with increasing age. As shown in **Figure 5L**, *colletotrichum* accounted for the largest proportion in 3Do, while *torula* and *cadophora* accounted for the largest proportion in 4Do. The proportion of *phaeosphaeria* increased with age in both *D. huoshanense* and *D. moniliforme*. In *D. officinale*, the proportion of *Phaeosphaeria* reached the largest in the second year. Overall, the community composition of the endophytes of the stems of the three 1–4-year-old *Dendrobium* species differed, with their fungal community composition being less complex than their bacterial community composition. At the same time, the endophyte community composition of the three *Dendrobium* species became more complex with increasing age.

### 3.5 Joint analyses of metabolome and microbiome

To further understand the regulatory relationship between endophytess and secondary metabolites and their effects on metabolism in *D. officinale*, D*. moniliforme*, and *D. huoshanense*, we analyzed the correlation between the microbiome and metabolome using a multi-omics analysis. We performed correlation analyses based on differentially expressed metabolites, differential flora (genus level), and chord diagrams of Spearman correlations between microbes and metabolites. Correlation analysis revealed potential interactions between endophytess and secondary metabolites in *Dendrobium* stems from different years. Based on the analysis of the transcriptome and metabolome above, we will focus on the correlation between endophytes and flavonoids.

**Figure 6:**
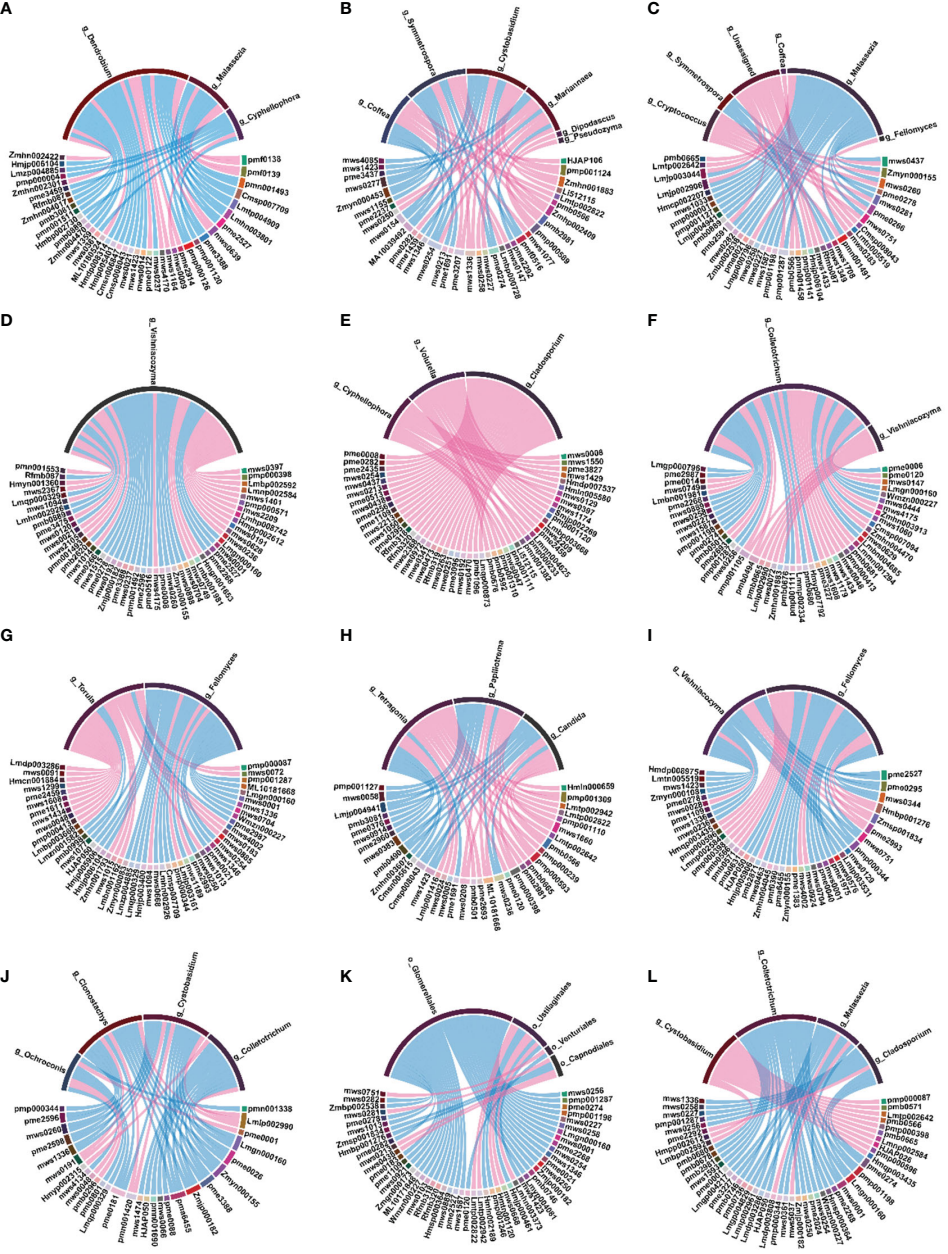
Spearman’s correlation chord diagram of differential microorganisms (bacteria) with DAMs at the genus level. **(A)** 1Dh vs 1Dm; **(B)** 1Dh vs 1Do; **(C)** 1Do vs 1Dm; **(D)** 2Dh vs 2Dm; **(E)** 2Dh vs 2Do; **(F)** 2Do vs 2Dm; **(G)** 3Dh vs 3Dm; **(H)** 3Dh vs 3Do; **(I)** 3Do vs 3Dm; **(J)** 4Dh vs 4Dm; **(K)** 4Dh vs 4Do; **(L)** 4Do vs 4Dm.

At the bacterial level, in 1Dh vs. 1Dm and 1Dh vs. 1Do, flavonoids (Genkwanin, Syringetin, Apigenin-8-C-Arabinoside, Tricin, Tricin-7-O-(2’’-Sinapoyl) glucoside, Apigenin-6,8-di-C-glucoside-4’-O-glucoside, Diosmetin-6-C-glucoside), flavonols (6-Hydroxykaempferol-7-O-glucoside) and flavonoid carbonoside (Apigenin-8-C-glucoside-7-O-(6’’-sinapoyl) glucoside, Isoorientin-7-O-glucoside, Hispidulin-8-C-glucoside) that showed significant positive correlation with *williamsia. Zymomonas* was significantly negatively correlated with several flavonoids, such as apigenin-7-O-glucoside (cosmosiin), kaempferol-3,7-di-O-glucoside, and luteolin-7-O-gentiobioside, in 1Dh vs. 1Dm. In 2Do vs. 2Dm, *lapillicoccus* was positively correlated with most flavonoids (rhamnocitrin, diosmetin, chrysoeriol, and tricin, among others). In 3Dh vs. 3Dm, *actinomycetospora* and *actinomycetospora* were positively associated with flavonols (Avicularin, Hyperin, Quercetin-7-O-(6’’-malonyl) glucosyl-5-O-glucoside). In 4Dh vs. 4Dm, *propionibacterium* was significantly negatively correlated with genkwanin, rhamnocitrin, diosmetin, chrysoeriol, hispidulin, and tamarixetin. Ninety percent of the bacteria significantly correlated with flavonoid levels were found to belong to the phyla actinobacteria and proteobacteria (**Figure 6**).

At the fungal level, *cyphellophora* was significantly negatively correlated with flavonoids in 1Dh vs. 1Dm. However, *cyphellophora* was significantly positively correlated with flavonoids in 2Do vs. 2Dm. *Vishniacozyma* was positively correlated with kaempferol-3,7-di-O-glucoside, luteolin-7-O-gentiobioside and apigenin in 2Dh vs. 2Dm. In 3Dh vs. 3Do, *vishniacozyma* was negatively correlated with 3’,4’,7-trihydroxyflavone, luteolin-3’-O-glucoside and genkwanin. *Colletotrichum* was negatively correlated with flavonoids in both 4Dh vs. 4Do and 4Do vs. 4Dm. Ninety-four percent of the fungi that were significantly associated with the levels of flavonoids belonged to ascomycota and basidiomycota (**Supplementary File Figure S4**).

## 4 Discussion

We detected differentially synthesized metabolites in the stems of *D. huoshanense*, *D. officinale*, and *D. moniliforme* from four different growth years. A total of 644 DAMs were identified, including 170 flavonoid metabolites, primarily flavonoids and flavonols. Notably, some important flavonoids, such as kaempferol, eriodictyol, luteolin, and tricetin, were synthesized at significantly higher levels in three- and four-year-old stems than in one- and two-year-old stems, regardless of the species; these have been reported to have high medicinal value. Kaempferol has been widely used in the treatment of multiple diseases because of its antioxidant and anti-inflammatory properties (Rajendran et al., 2014). Eriodictyol exhibits significant antioxidant (Rossato et al., 2011) and anti-inflammatory properties (Lee, 2011). Luteolin exhibits antioxidant, anti-inflammatory, anti-allergic, and anticancer properties (Seelinger et al., 2008; Imran et al., 2019; Gendrisch et al., 2021). Tricetin exhibits anti-inflammatory properties, in addition to inhibiting the proliferation of human breast cancer cells by blocking cell cycle progression and inducing apoptosis (Hsu et al., 2009; Sun et al., 2019). The accumulation of active ingredients is a potential factor affecting the complete utilization of medicinal plants, and it is of great significance to understand the synthetic mechanism. In summary, multiple flavonoids showed significant accumulation in the stems of *D. huoshanense*, *D. officinale*, and *D. moniliforme*, indicating that flavonoids play an important role in the growth of these plants. Therefore, the stems of *D. huoshanense*, *D. officinale*, and *D. moniliforme* have significant medicinal value.

Furthermore, we constructed a regulatory network for flavonoid biosynthesis in *D. huoshanense*, *D. officinale*, and *D. moniliforme* stems to concisely visualize the role of genes involved in flavonoid synthesis in this pathway. Our data showed 37 DEGs involved in flavonoid biosynthesis in six comparison groups, including *PAL, C4H, 4CL, CHS, CHI, F3H, F3’H, F3’5’H* and *FLS* genes. In addition, we identified key metabolites and genes by analyzing metabolite-gene interactions in the flavonoid biosynthetic pathway. *CHS* was reported to be a key enzyme in the flavonoid biosynthetic pathway, involved in the upstream step of the pathway (Winkel-Shirley, 2001), and is the first rate-limiting factor in the flavonoid biosynthetic pathway (Austin and Noel, 2003). *CHS* also drives the production of phytoalexins in plants in response to different forms of stress and is involved in a series of defense responses (Dao et al., 2011; Ye et al., 2017). Notably, in plants such as *Citrus unshiu* (Wang et al., 2010), *Grewia asiatica* (Wani et al., 2017), *Syringa oblata* (Wang et al., 2017), and *Ginkgo biloba* (Xu et al., 2007), *CHS* expression levels were reported to be highly correlated with the accumulation of flavonoids. Consistent with the results of previous studies, the pathway diagram clearly showed that the expression of *CHS* promoted the accumulation of flavonoids. The expression levels of two *CHS* genes (LOC110115249 and LOC119195073) were upregulated in the third and fourth years of *D. moniliforme* growth, and the corresponding flavonoid naringin chalcone also accumulated to a certain extent. Naringin chalcone has received widespread attention owing to its numerous pharmacological activities. Reportedly, it has antibacterial, anti-cancer, anti-tumor (Zhang et al., 2016b), anti-inflammatory (Ur Rashid et al., 2019), anxiolytic and anticonvulsant (Ferreira et al., 2021) properties, with high applicability and market value. Therefore, the accumulation of flavonoids in *D. moniliforme* may be closely related to the expression of *CHS*.

The literature has demonstrated that *curtobacterium* represents a group of Gram-positive phytopathogens, primarily associated with infections in legume and sugar beet (Chen et al., 2007; Osdaghi et al., 2015). The pathogenicity of *curtobacterium* in different host plants is different. *Curtobacterium* can cause bacterial wilt in legumes, but it has not been pathogenic to solanaceae (Osdaghi et al., 2018). Currently, there is no scientific evidence indicating the pathogenicity of *curtobacterium* towards *Dendrobium*. However, it is crucial to exercise caution and implement effective control measures in order to safeguard the quality of the harvest of *Dendrobium*. Through the previous analysis, we found that with the increase of growth years, the proportion of some pathogenic fungi gradually increased. *Phaeosphaeria* is a pathogen of leaf spot disease (Do Amaral et al., 2005). At present, leaf spot is mainly reported in *D. officinale* (Cao et al., 2022). It is noteworthy that *phaeosphaeria* accounted for a larger proportion in the *D. moniliforme* and *D. huoshanense* in this study. Therefore, cultivators of *D. moniliforme* and *D. huoshanense* need to pay extra attention to leaf spot, especially if they have been planted continuously for many years. *Colletotrichum* is the cause of anthrax, a common pathogen found in vegetables and fruits (Liu et al., 2016). Sarsaiya et al. isolated five endophytic bacteria from *D. nobile* and found that *colletotrichum* was highly pathogenic and even caused host death by studying the pathogenicity of these five bacteria (Sarsaiya et al., 2020). Our results show that *colletotrichum* accounts for a significant proportion of the composition of the fungal community of triennial *D. officinale*. Therefore, the growth period of *D. officinale* should not be too long, otherwise it will accumulate a large number of harmful endophytic bacteria, which will lead to its quality decline.

Lastly, based on the results of the joint metabolome and microbiome analysis, the highest correlation between microorganism abundance and flavonoid levels was observed in bacteria, specifically in actinobacteria and proteobacteria. The abundances of ascomycota and basidiomycota showed the highest correlation with flavonoid levels. Xie et al. investigated the metabolites, transcriptome, and microbiome of 1-35-year-old poplar roots and found that the abundance of actinobacteria was strongly correlated with flavonoid biosynthesis with the increase in tree age (Xie et al., 2023). The study conducted by Natsagdorj et al. utilizing HPLC analysis revealed that endophytic actinobacteria, which were isolated from desert plants, demonstrated the ability to synthesize flavonoids and phenolic compounds (Natsagdorj et al., 2021). The findings of these studies suggest a strong correlation between actinobacteria and the biosynthesis of flavonoids in the stems of *Dendrobium*. In our previous analysis, we observed a significant correlation between *williamsia*, *lapillicoccus*, *actinomycetospora* and *actinomycetospora* with flavonoid synthesis. However, there is currently no existing literature on the correlation between these bacteria and flavonoid biosynthesis. We hypothesize that these microorganisms have potential flavonoid production capabilities, but further experimental verification is needed.

## 5 Conclusion

We used multi-omics to analyze the different growth years of three types of medicinal dendrobium and found that the flavonoid content of *Dendrobium huoshanensis* and *Dendrobium moniliforme* increased with the increasing years. Furthermore, we analyzed the endophytes that affect medicinal ingredients and found that as the cultivation years increased, the pathogenic microbial in the stems of the three types of *Dendrobium* also gradually increased. Although the four-year-old *Dendrobium* also had higher flavonoids, the pathogenic microbial also increased. Therefore, after comprehensive consideration, it is recommended to harvest flavonoids in the third year. And based on the results of previous analysis, it was also found that there is a close connection between actinobacteria and the synthesis of flavonoid compounds in *Dendrobium* stems, which provides some valuable references for our subsequent research.

## Data availability statement

The datasets presented in this study can be found in online repositories. The names of the repository/repositories and accession number(s) can be found in the article/**Supplementary Material**.

## Author contributions

YY: Conceptualization, Investigation, Writing – original draft. JZ: Software, Writing – original draft. XW: Data curation, Supervision, Validation, Conceptualization, Writing – original draft. RZ: Data curation, Methodology, Writing – original draft. WX: Supervision, Writing – review & editing. SL: Conceptualization, Supervision, Writing – review & editing.
