# Supplementary material for: Multi-omics profiling reveal responses of three major Dendrobium species from different growth years to medicinal components
Source: Front Plant Sci. 2024 Feb 23;15:1333989. doi: 10.3389/fpls.2024.1333989 (PMC10920241; doi:10.3389/fpls.2024.1333989)
Supplement: Supplementary file 1 [file DataSheet_1.docx]

Table S1. Estimate of alpha-diversity of endophytic bacteria from the stems of three kinds of *Dendrobium* of 1-4-year-old.

|  | Shannon | Chao1 | ACE |
| --- | --- | --- | --- |
| 1Dh | 2.47 ± 1.35ab | 426.08±190.75ab | 422.04±194.80ab |
| 2Dh | 2.49 ± 1.29ab | 433.06±138.63ab | 417.52±137.99ab |
| 3Dh | 3.61 ± 1.73ab | 889.69±524.44ab | 862.00±493.03ab |
| 4Dh | 4.56 ± 0.12ab | 1108.40±149.71a | 1089.26±153.88a |
| 1Dm | 1.69 ± 0.20b | 371.34±32.71b | 360.69±22.20b |
| 2Dm | 4.85 ± 0.10a | 1037.39±155.08a | 1029.64±132.83a |
| 3Dm | 3.97 ± 0.39ab | 866.95±190.40ab | 864.92±180.56ab |
| 4Dm | 4.18 ± 0.38ab | 904.08±152.86ab | 892.97±170.98ab |
| 1Do | 1.61 ± 0.39b | 301.35±62.70b | 311.09±69.45b |
| 2Do | 2.89 ± 2.02ab | 475.13±405.42ab | 473.95±384.34ab |
| 3Do | 3.42 ± 1.15ab | 598.31±163.61ab | 585.38±171.40ab |
| 4Do | 4.51 ± 0.18ab | 684.60±116.39ab | 672.38±119.60ab |

Table S2. Estimate of alpha-diversity of endophytic fungi from the stems of three kinds of *Dendrobium* of 1-4-year-old.

|  | Shannon | Chao1 | ACE |
| --- | --- | --- | --- |
| 1Dh | 1.15±0.34a | 73.94±1.17a | 75.62±3.70a |
| 2Dh | 1.16±0.40a | 54.08±10.75a | 57.65±13.16a |
| 3Dh | 1.45±0.97a | 108.38±44.02a | 107.84±43.16a |
| 4Dh | 1.63±0.21a | 105.68±11.34a | 105.27±10.83a |
| 1Dm | 1.81±0.55a | 81.93±17.25a | 84.24±15.72a |
| 2Dm | 1.36±0.45a | 60.78±4.94a | 61.81±4.83a |
| 3Dm | 1.96±0.06a | 94.03±4.18a | 95.70±3.67a |
| 4Dm | 1.88±0.89a | 108.00±55.97a | 107.80±55.89a |
| 1Do | 1.31±0.38a | 141.99±18.87a | 142.63±20.12a |
| 2Do | 1.61±0.74a | 78.51±44.69a | 78.79±44.03a |
| 3Do | 1.27±0.31a | 110.59±52.53a | 112.25±51.94a |
| 4Do | 1.79±0.48a | 113.71±22.73a | 113.32±18.73a |

Figure legends

Figure S1 Multi-group difference scatterplot for displaying DEGs across multiple comparison groups.

Figure S2 Volcano plots of the DAMs in different comparisons. The green dots mean significantly down-regulated genes, the red dots mean significantly up-regulated genes, and the grey dots mean not-significant DAMs. (a) 1Dh vs 1Dm; (b) 1Dh vs 1Do; (c) 1Do vs 1Dm; (d) 2Dh vs 2Dm; (e) 2Dh vs 2Do; (f) 2Do vs 2Dm; (g) 3Dh vs 3Dm; (h) 3Dh vs 3Do; (i) 3Do vs 3Dm; (j) 4Dh vs 4Dm; (k) 4Dh vs 4Do; (l) 4Do vs 4Dm.

Figure S3 The KEGG pathway analysis of DAMs. (a) 1Dh vs 1Dm; (b) 1Dh vs 1Do; (c) 1Do vs 1Dm; (d) 2Dh vs 2Dm; (e) 2Dh vs 2Do; (f) 2Do vs 2Dm; (g) 3Dh vs 3Dm; (h) 3Dh vs 3Do; (i) 3Do vs 3Dm; (j) 4Dh vs 4Dm; (k) 4Dh vs 4Do; (l) 4Do vs 4Dm.

Figure S4 Spearman’s correlation chord diagram of differential microorganisms (fungi) with DAMs at the genus level. (a) 1Dh vs 1Dm; (b) 1Dh vs 1Do; (c) 1Do vs 1Dm; (d) 2Dh vs 2Dm; (e) 2Dh vs 2Do; (f) 2Do vs 2Dm; (g) 3Dh vs 3Dm; (h) 3Dh vs 3Do; (i) 3Do vs 3Dm; (j) 4Dh vs 4Dm; (k) 4Dh vs 4Do; (l) 4Do vs 4Dm.


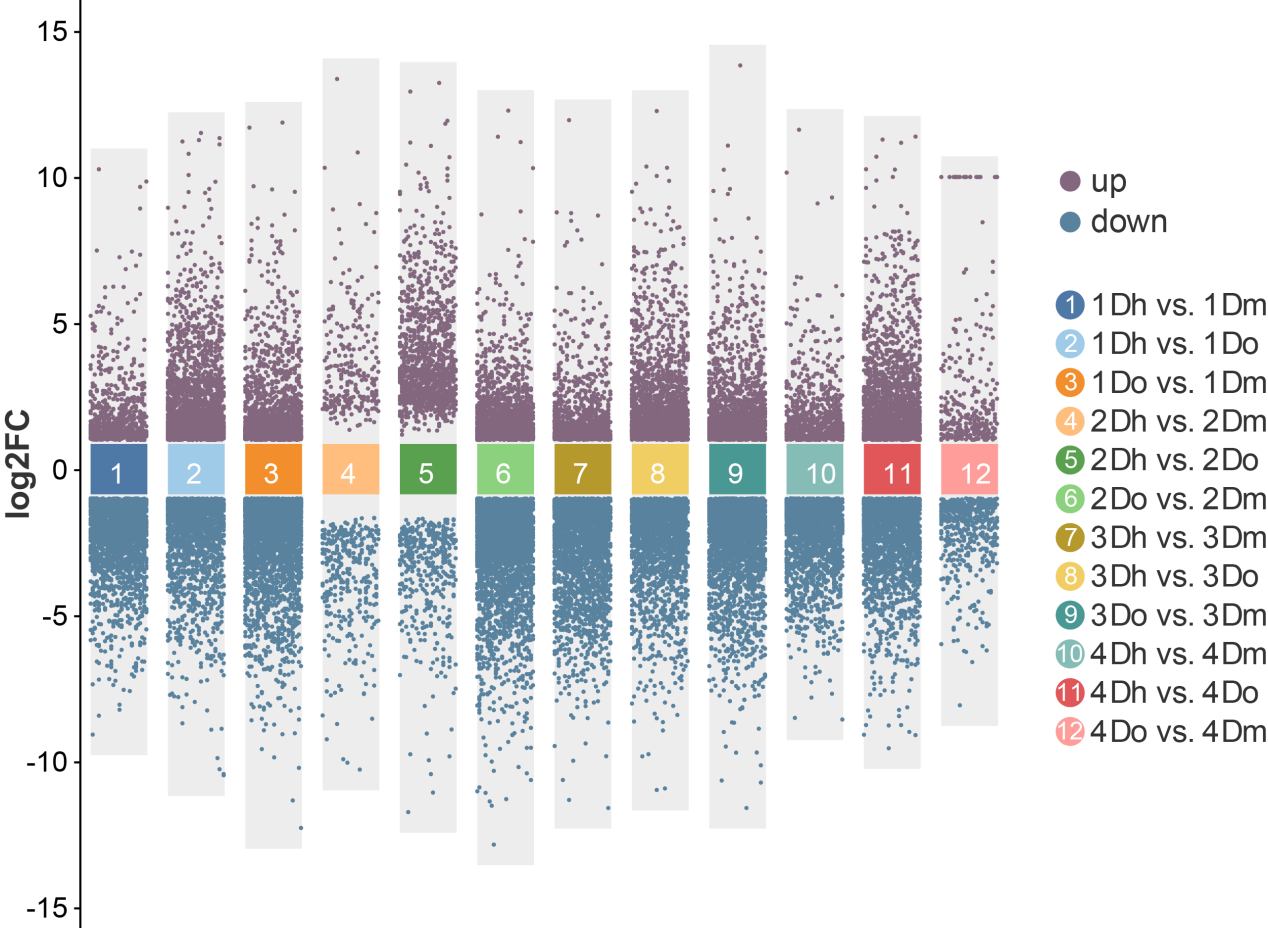


Figure S1


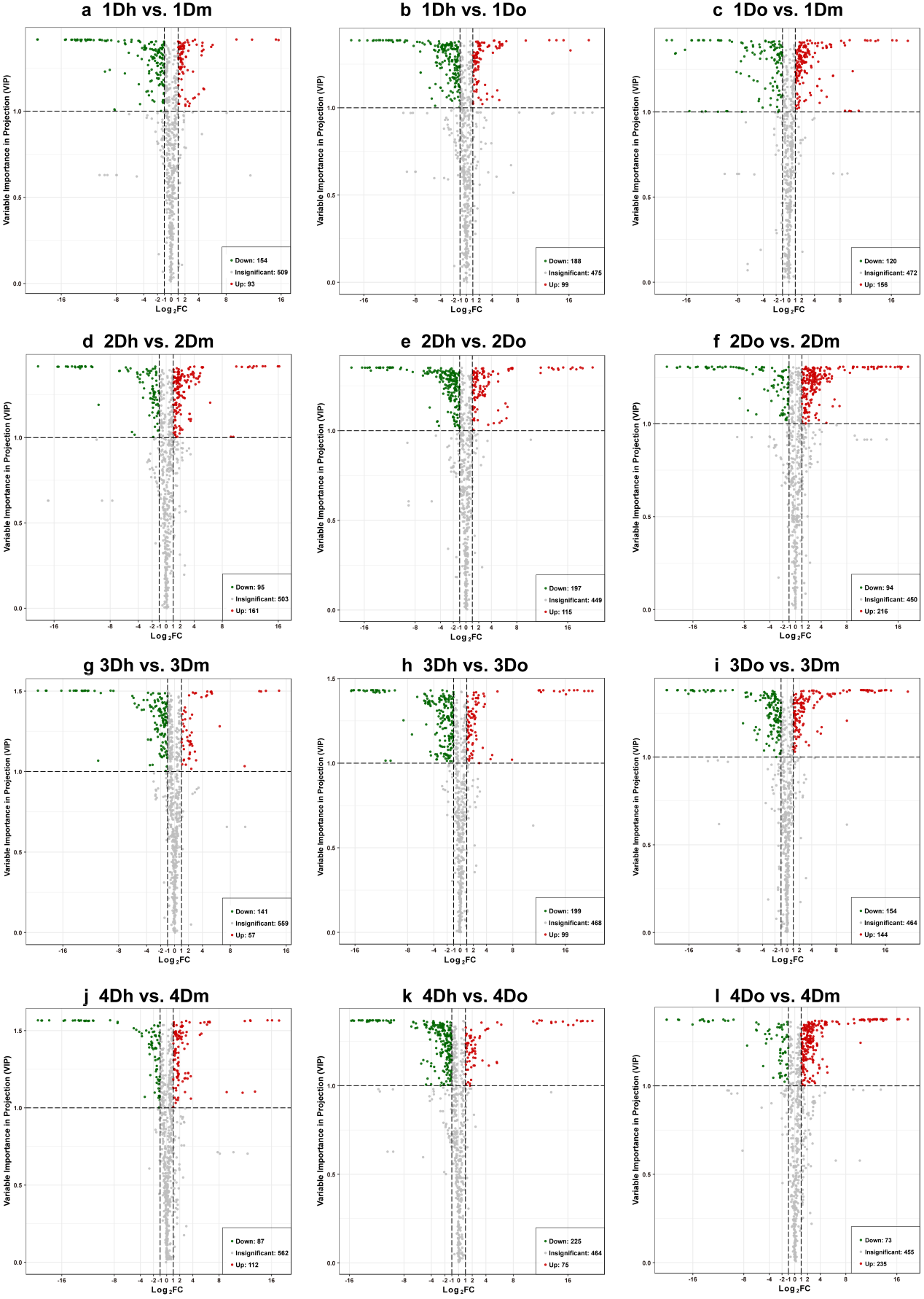


Figure S2


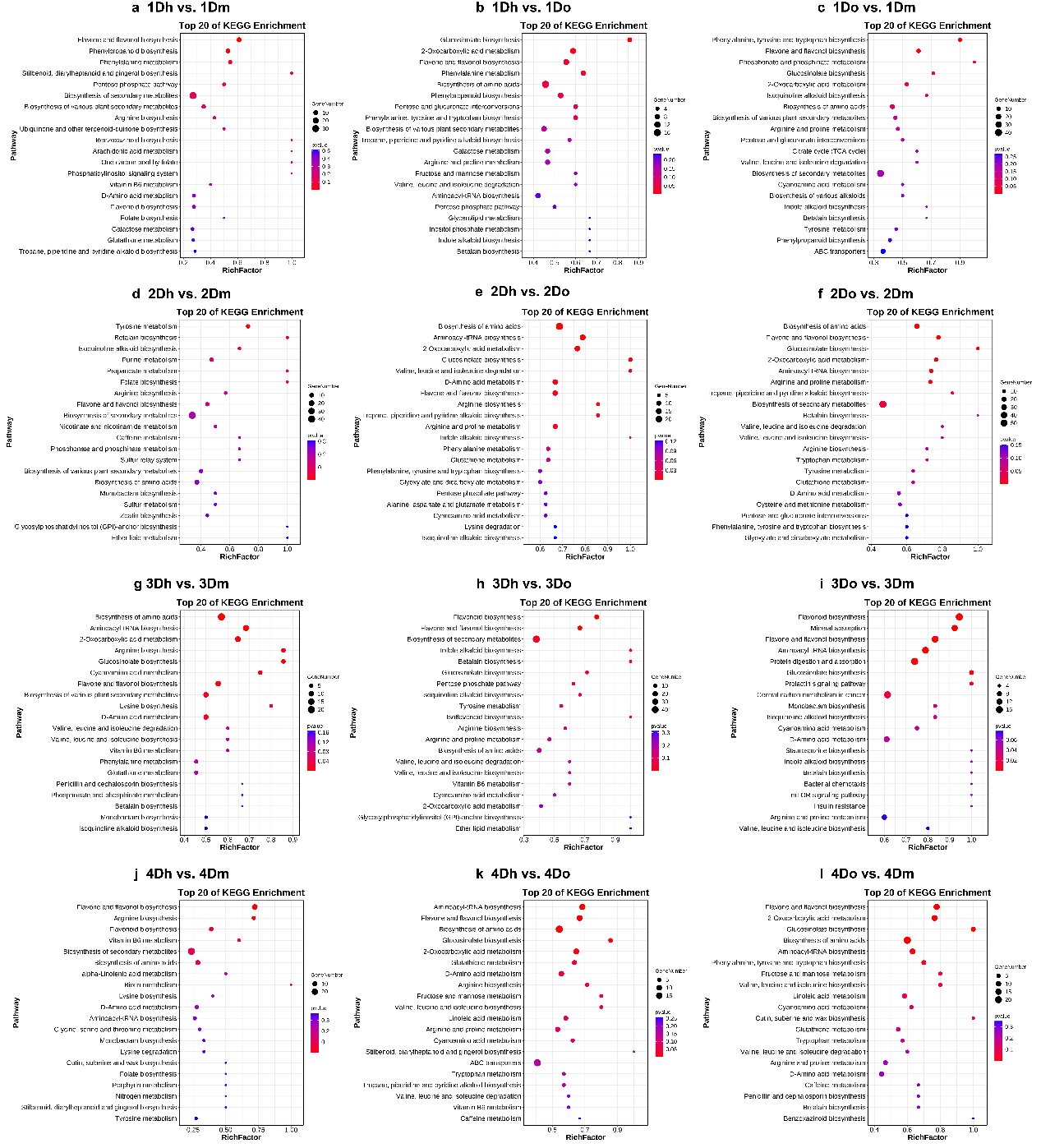


Figure S3


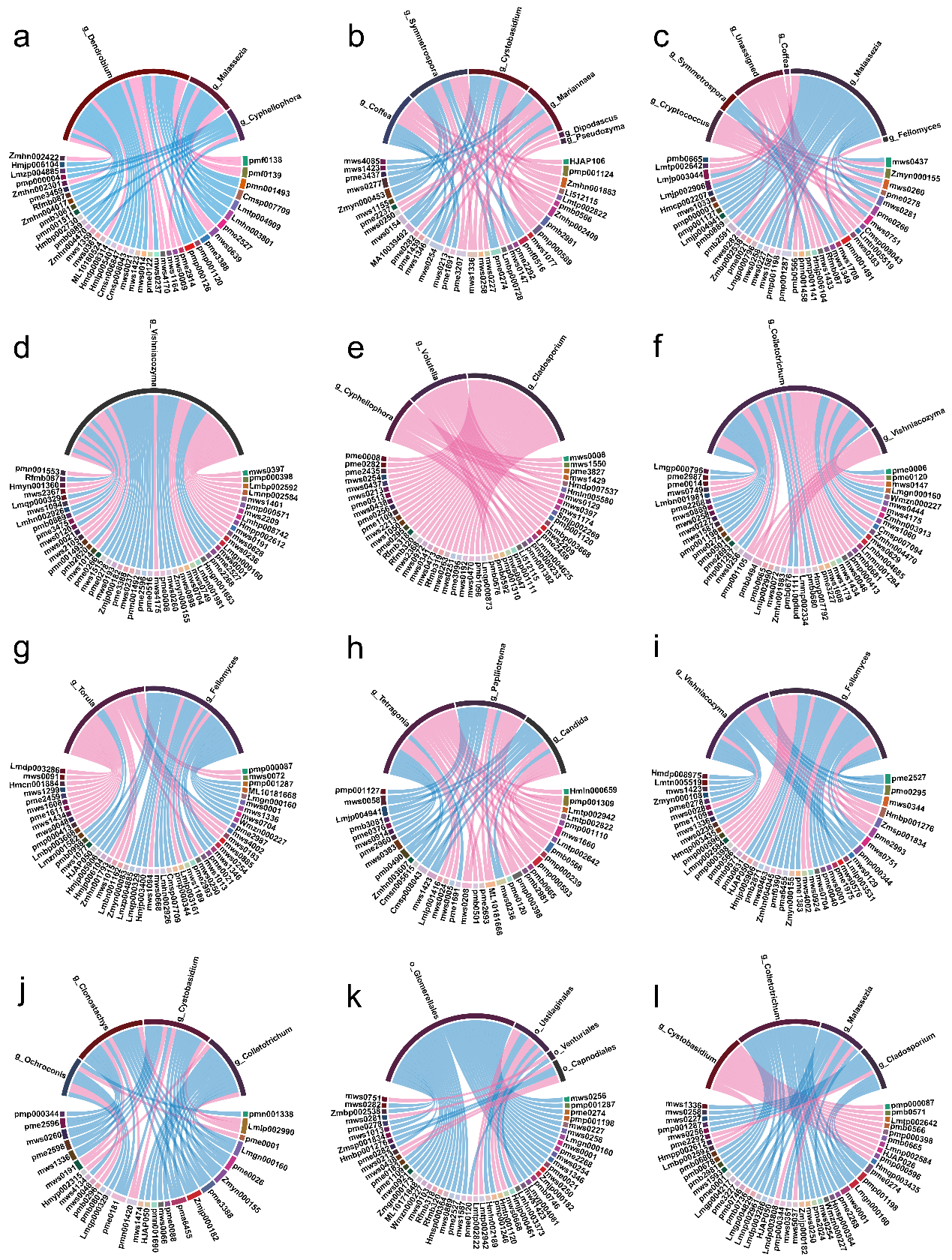


Figure S4
